# Southern Medical Reports

**Published:** 1851-03

**Authors:** 


### ARTICLE II.

*Southern Medical Reports*, Edited by E. D. FENNER, M. D., of New Orleans, Volume 1st, 1849.

(CONCLUDED.)

Our notice of this volume in the last number of this Journal was confined exclusively to those papers which related to the Topography and Meteorology in connection with the diseases of particular localities. Two papers of the same character remain, which were not particularly noticed in the previous number. The first purports to be a "General Report on the Topography, Meteorology and Diseases of Jackson, the Capitol of Mississippi, by S. C. Farrar, M. D.," and the second, "Report on the Topograpy of San Antonio, and the Epidemic Cholera that prevailed there in the spring of 1849, by J. J. B. Wright, Surgeon U. S. Army."

The Topographical part of Dr. Farrar's paper, is comprised in little more than one page and a half, furnished to him by his friend Rev. A. Morris. Following this is a Meteorological table obtained from Mr. and Mrs. Oakley, Principals of the Oakland Institute, and nearly two pages of "*observations*" on the weather of each month. The following are specimens of these observations, viz:

"February.—A few of the earlier days of this month were so genial, that butterflies came out, and mocking birds began to sing. Then on the 19th the thermometer fell to 19 deg. This killed peas and many other early vegetables."

"June.—Gave a continuation of the showery cloudy weather of May. The whole quantity of rain was not exceedingly great, but falling on fourteen different days it produced much inconvenience to the planter."

There is no mention made of the diseases which prevailed during each month, as influenced by the meteorological changes, or the special Topography of each locality; but all the references, whether to soil, water, or air, are so general as to admit of no practical application. Indeed, for all practical purposes, the whole of this part of Dr. Farrar's paper, might as well have been omitted. In this respect Dr. Wright's paper is still more defective. The following is the sum total of his account of the Topography of San Antonio.

"The city of San Antonio is situated in the midst of a valley, of unequal dimensions, through which, in a general direction runs the serpentine river of that name, having its origin in several beautiful springs four or five miles northeast of the city. The Rio San Pedro also takes its rise in a spring two miles north of the town, and running in a direction nearly parallel with the San Antonio, it skirts the city on its western border, and forms a junction with the latter two miles below the city. A large portion of the town is in proximity to San Antonio, in some parts of its course as it runs west and south, and west again, and in its meanderings leaves the precincts of the city, pursuing a course east of south. The San Antonio is a rapid stream, having a celerity of three or four miles an hour, varying in depth from three to six or seven feet, and averaging fifty feet in width.

The San Pedro is less by one half than the San Antonio, but resembles it in the qualities of its water, and in general character. The river water is pretty strongly impregnated with lime, but is agreeable to the taste, and is almost exclusively used for culinary purposes and all other purposes. Stories were told and got admission into the newspapers, representing the water as having acquired a fœtid smell, and changed appearance during the prevalence of the epidemic. This statement seems founded in error, at least the alleged fact is not in accordance with my observation, and a constant and exclusive use of the water authorizes me to speak positively on the subject. The superstratum included between the rivers, and comprising the limits of the city, consists of a dark rich mould, vegetable debris, with large admixture of disentangled limestone, rendering the mud after rains almost as viscid as bird-lime.

It is remarkably fertile when well watered, but without irrigation in seasons of drought, the surface acquires a stiffened exsiccated condition, which unfits it for cultivation, Almost the only rock in the vicinity of San Antonio, consists of rotten and fossiliferous lime-stone, much of which has become disintegrated on and near the surface, rendering the soil in some places almost incapable of sustaining vegetable life."

We have thus quoted the whole of Dr. Wright's remarks on Topography, first, because it serves as an example illustrating the *general* and *indefinite*, and consequently valueless, characacter of too many such reports; and second, because without the report itself before them, our readers might deem our subsequent remarks unjust or hypercritical. When we find a paper headed, "Report on the Topography and Diseases" of any given locality we very naturally look for such an exposition of the former, as will show its influence, direct and indirect, on the prevalence, specific character, and results of the latter. To this end, we must be informed, first, of the surface of the district included ; its altitude both absolute and relative to surrounding districts ; the water resting on it, whether moving or stagnant ; the depressions and elevations, and their character for dryness or moisture ; second, the composition of the soil, sub-soil, and underlaying strata of rocks ; the relative position of such strata in reference to their permeability to water, thereby showing whether the surface water readily drains off, or is retained by horizontal strata of rocks or tenacious clay, until re-evaporated into the atmosphere, carrying along with it, perhaps, more or less deleterious matter from the soil ; third, the qualities of the water, both that naturally found in the soil, and that used by the inhabitants ; and also whether such qualities vary with the varying temperature of the seasons ; fourth, the number, character, and habits of the inhabitants ; their positions relative to standing waters, low grounds, prevailing winds, &c. ; and if a city is included, we must be informed in regard to the houses and streets, in reference to cleanliness, ventilation, drainage, and light ; and fifth, the comparative prevalence and severity of particular diseases, in special localities or streets ; among particular classes of the people ; and at particular seasons, having reference to cold, heat, dryness, winds, character of food, water, &c. With such information before us, we can judge of the influence of local causes on the prevalence and character of diseases—a subject of the greatest importance and most intense interest ; a subject indeed, which must be carefully and most patiently studied, not in loose generalities, but with the greatest minuteness, before etiology can be stripped of its present vagueness and made to approximate a satisfactory degree of clearness and certainty. The reader will see, however, that Dr. Wright has entered upon no such exposition of the Topography of San Antonio. He has not even told us the *number* of its inhabitants, much less their habits and modes of life; nor in the subsequent part of his paper, has he made even a remote allusion to the influence of any local conditions or circumstances, on the developement and character of the diseases of which he speaks. Very nearly the same remarks are applicable to the paper of Dr. Farrar, and indeed to all the others in this volume, except those of Drs. Pendleton and Fenner. We will not characterize all such reports as entirely useless in their relation to Medical Topography, but we must style them very unsatisfactory, and unworthy the name they bear. But we shall look to Dr. Fenner's project, in publishing an annual volume of original papers, with no little expectation of seeing it produce a marked improvement in this respect.

The papers to which we have now alluded, as containing more or less of Topography and Meteorology, make up about one third of the entire volume. Of the remaining papers five are devoted to the consideration of Epidemic Cholera, written by Drs. C. H. Stone of Natches, C. S. Magoun of Natches, Lewis Shanks of Memphis, N. S. Jarvis of Texas, and the Editor, Dr. Fenner of New Orleans. These papers make up seventy-eight pages, and though interesting and valuable, as embodying the opinions and experience of individuals, yet we find nothing in them in relation to the causes, pathology, or treatment of that dreaded pestilence which has not already found its way into the Medical Journals, and been extensively distributed through the profession.

Indeed, the papers before us present the usual diversity of views in regard to all these points. Yet their differences are more apparent than real, relating more to the detail of means for the accomplishment of certain objects, than to the objects themselves. It is true that some like Dr. Wright, of the U. S. Army, distrust the utility of all medicine, and declare, as they allege, "in a spirit of honesty and candor, that I, (they,)

very much doubt, in the language of Prof. Watson, 'if the aggregate mortality from Cholera, (fully developed) was in any way disturbed by our craft.'"

The following remarks of Dr. Fenner on this point, are so just, and so well calculated to inculcate right feelings, and encourage right action, that we quote them at the risk of being tedious. He says:

"We are not among those who admit they *know nothing about cholera or its remedies.* On the contrary, we contend that the profession knows a great deal about both. We do not know what the *remote cause* of cholera is; neither do we know what are the *remote causes* of fever, dysentery, measles, &c.; but we are well acquainted with the *effects* of all these causes. We know how cholera attacks people, how the disease progresses in its destructive march, how it terminates in convalescence or death; and also what remedies will cure the great majority of cases, if applied judiciously and at the proper time. Is all this knowledge worth nothing? If so, we had as well confess our ignorance of all diseases, and, with folded arms, resign ourselves and the community to *inexorable fate!* But it is not so. Our Omnipotent and Merciful Creator has not left us in this helpless and powerless state. * * * There can be but little doubt that at least *eight tenths of the victims of cholera in New Orleans have died unnecessarily*; i. e., they have been lost on account of their neglect of the plainest dictates of prudence and common sense. Ought this to be charged to the discredit of the medical faculty? Or ought we to confess that so many people have died of cholera because we did not know how to treat the disease? Certainly not. We do know how to treat it, and as the best evidence of the fact, we have seldom failed to cure our patients, if called in before they are *beyond the curable stage.*"

These sentiments fully accord with my own experience during two successive years of practice in the treatment of epidemic cholera. Thus, during the past summer and autumn

96 well marked cases of cholera occurred in my private practice, a majority of whom were among our Norwegian and Swedish populations. Of these 96, *twenty-four* died. Of these 24, *eleven* were in complete collapse, I might say, in *articulo mortis*, before medical aid was either sought for or obtained. *Five* others died from consecutive affections, occasioned by neglect or marked imprudence ; leaving only *eight* deaths under fair and reasonable opportunity for treatment. And such will always be the result when judicious and rational means are faithfully applied at any stage of the disease, prior to that loss of capillary action and organic nervous sensibility, which render the system incapable of receiving the impressions of medicinal agents. But in all populous communities, there are thousands who never think of sending for medical aid until they have exhausted a list of *nostrums*, or spent the whole curable period of the disease in *toping whiskey* or *brandy*, and then because the physician can do little else than arrive in time to see them die, it is gravely concluded that he knows little or nothing about the disease. A conclusion as absurd as it is discouraging to all rational inquiry. Time and space however, admonish us to hasten on. The remaining papers of most interest to the profession, are: "Special Report on the Fevers of New Orleans, particularly the Yellow Fever of 1849, by the Editor." "Reports on the origin and sanitary condition of the Orphan Asylums of New Orleans and La Fayette." And, "Observations on the Fever which is developed in the City of Charleston, after exposure to the country air, during the summer and autumn, and hence called country fever, by Thos. Y. Simmons, M. D." This last paper is one of much interest. It was first published in the Charleston Medical Journal and Review for Sept. 1849, and has consequently been sometime before the profession. The other two appear for the first time in the volume before us, and are not only interesting and important, but must be read throughout to be properly appreciated. The Orphan Asylums alluded to in tbe second paper, are the Poydras Female Orphan Asylum; the New Orleans Female Orphan Asylum; and the Male Orphan Asylum of La Fayette. The first contained at the time of the report about 150 children, and though a protestant institution, its benefits are extended alike to all classes; the second is under the care of the Sisters of Charity, and numbers about 200 inmates. While the cholera was raging violently in the city and the immediate vicinity of the Asylum, the attending physician directed special attention to the diet of the children, and caused each to take a small quantity of a solution of common table salt every morning. No case of cholera originated in the institution during the year.

The Male Orphan Asylum was founded in 1824, and contained at the close of the year 1849, eighty-six boys. Thus making a total in the three asylums of 436. These institutions seem to be admirably managed, and are truly an honor to their generous founders and patrons. To the practical reader, perhaps no part of the whole volume is more interesting than Dr. Fenner's paper on the Fevers of New Orleans. Contrary to the generally received opinions of the profession, Dr. Fenner denies the *sui genius* character of Yellow Fever, and regards it simply as a variety of the ordinary Intermittent and Remittent forms of Fever. Thus he says: "Cases are seen every sickly year, commencing as intermittent or mild remittent, and wanting those strongly marked diagnostic symptoms which have been said to distinguish yellow fever, yet which, if neglected or mal-treated, terminate in *hemorrhage* and *black vomit.* In these cases, the advocates of the *specific* character of Yellow Fever, contend that the patients contract a *new* and *different* disease; but we think improperly. *We believe it is all the same disease, differing only in grade and stage.*" Again he says: "Seeing then that all the forms of idiopathic fever met with in this locality, *prevail together*, and are frequently seen to *interchange or run into each other*, we are irresistably led to the conclusion that they are merely *modifications of disease*

*springing from one and essentially the same general remote cause.* * * * * Our position is, that yellow fever is only one of the forms of endemic fever, (malarious if you will,) which derives its characteristic features from the locality and attendant circumstances where it prevails. The fevers of the country cause death by inflammation of the brain, or the gastro-intestinal canal, or by that strange lesion of the nervous system which is called congestion. The fevers of the city produce such an alteration of the blood and the solids as leads to fatal hemorrhage and jaundice." These views of Dr. Fenner accord very nearly with those promulgated by Dr. Rush, and are entertained by a few of the ablest members of the profession both in Europe and America; and we have little doubt of their correctness. Indeed, we are more than half convinced that the same comprehensive view of all the parts in relation to ordinary cholera morbus and epidemic cholera, will lead to the conclusion that they bear the same relation to each other, as is here set forth in regard to intermitting, remitting and yellow fevers. We think there is far too great a tendency in the profession to look after distinct unrecognized and unrecognizable or supposititious remote causes, instead of investigating with patience and accuracy the *local* influencies and agencies which modify, and often control the most malignant forms of disease. In regard to the *treatment* of yellow fever, Dr. Fenner is a strong advocate of what he terms the "*abortive method of quinine.*" He says: "When called to a case within twenty-four or thirty-six hours of the attack, we seldom failed to cut short the fever by *large doses* of the sulphate of quinine in combination with opium or morphia, frequently followed by a little blue mass or calomel. Our usual mode of proceeding in this stage is, to order at first, a hot, mustard foot bath, and a purgative enema—then give to an adult 20 or 30 grains of quinine, with 25 or 30 drops of laudanum, or one or two grains of opium, or the fourth of a grain of sulphate of morphia, *at one dose.* This would generally reduce the vascular and nervous excitement completely in a few hours, throw the patient into a profuse perspiration, relieve all pain and produce sleep. The bowels were kept open by some gentle means, and more or less quinine was repeated as occasion required. We recollect of but one fever patient that required cupping, and we did not have a single one bled from the arm." It will be remarked that this treatment has reference to the early stage of the disease. "In the advanced stages," says Dr. Fenner, "it is altogether a different affair—*organic lesions have then taken place and the blood is altered.*"

There is one other paper in this volume of high scientific merit and local interest. It is entitled "A chapter on the Hydograpy of the Misssissippi River, by Caleb G. Forshay, A. M., Civil Engineer." It does not, however, come within our province to analyze it. We have now glanced over the contents of this first volume of the Southern Medical Reports, and though we have found some fault with the character of some of the papers contained therein, and might justly have found more with the style of others, yet we deem it a most valuable volume, and we commend the enterprise of its able Editor to the patronage of the whole profession. N. S. D.

Chicago, Feb. 25th 1851.

